# Incidental Diagnosis of Serrated Polyposis Syndrome of the Small and Large Bowel Treated Endoscopically and Surgically: A Case Report

**DOI:** 10.7759/cureus.108912

**Published:** 2026-05-15

**Authors:** Hind Joumaa, Fatima Alsalman, Lama Chouman, Mohammad Siblini, Walid Nasreddine, Zeena Haress, Ibtisam Al jamal, Mohamad El Haress

**Affiliations:** 1 Department of Gastroenterology and Hepatology, Makassed General Hospital, Beirut Arab University, Beirut, LBN; 2 Department of Gastroenterology and Hepatology, Makassed General Hospital, Beirut, LBN; 3 Department of General Surgery, Makassed General Hospital, Beirut, LBN; 4 Department of Anatomy, Physiology, and Cell Biology, American University of Beirut, Beirut, LBN; 5 Department of Neuroscience, British Neuroscience Association, Brighton, GBR; 6 Department of General Surgery, Makassed General Hospital, Beirut Arab University, Beirut, LBN; 7 Department of Surgery, American University of Beirut Medical Center, Beirut, LBN

**Keywords:** colorectal cancer (crc), colorectal polyposis syndrome, screening endoscopy procedures, serrated polyposis syndrome (sps), small intestine neoplasm

## Abstract

Colorectal serrated polyps are now recognized as an important pathway in colorectal carcinogenesis. An association between serrated polyposis syndrome (SPS) and duodenal serrated lesions sharing a similar immunophenotype in both the colon and duodenum has been reported in the literature.

We present a case of a 38-year-old male who presented in June 2023 with abdominal pain and fever. Initial investigations, including multiple computed tomography scans of the abdomen and pelvis, revealed an appendiceal abscess and small bowel intussusception involving two segments of the jejunum without evidence of obstruction, along with multiple polyps throughout the small and large bowel suggestive of an intestinal polyposis syndrome.

Further evaluation with colonoscopy, esophagogastroduodenoscopy, and magnetic resonance enterography confirmed the presence of numerous polyps of varying sizes involving the duodenum, jejunum, ileum, and all segments of the colon. The initial differential diagnosis included familial adenomatous polyposis, Peutz-Jeghers syndrome, and PTEN hamartoma tumor syndrome.

The patient subsequently developed complicated small bowel obstruction due to intussusception and underwent open small bowel resection. Multiple intestinal polyps were identified, and histopathological examination confirmed the diagnosis of SPS.

At a later stage, the patient was planned for subtotal colectomy, preceded by elective endoscopic polypectomy, which achieved clearance of the left colon up to 20 cm from the anal verge. Gross examination of the colectomy specimen revealed more than 20 large polyps measuring up to 4 cm. Histopathological analysis demonstrated colonic serrated polyposis without evidence of invasive malignancy, wall infiltration, or lymph node involvement, with clear surgical margins.

## Introduction

Colorectal serrated polyps (SPs) were first described by Longacre and Fenoglio-Preiser in 1990 [1]. These lesions are histologically classified into three main subtypes: hyperplastic polyps (HPs), traditional serrated adenomas (TSAs), and sessile serrated polyps (SSPs).

In 2019, the World Health Organization (WHO) updated the diagnostic criteria for serrated polyposis syndrome (SPS), which include (i) the presence of five or more serrated lesions proximal to the rectum, all measuring ≥5 mm, with at least two measuring ≥10 mm, or (ii) more than 20 serrated lesions of any size distributed throughout the colon, with at least five located proximal to the rectum [2].

Although adenomatous polyps remain the most common precursors of colorectal cancer (CRC), approximately 30% of CRC cases are thought to arise via the serrated pathway, particularly from SSPs [3]. Despite being underdiagnosed, SPS is the most common colorectal polyposis syndrome and is associated with an increased risk of both the development and incidence of CRC [3].

Close and regular surveillance is essential due to the risk of malignant transformation. Endoscopic resection of serrated lesions, combined with scheduled follow-up, represents the cornerstone of management. However, surgical intervention may be required when endoscopic treatment is not feasible or unsuccessful.

Small intestinal involvement in SPS is rare, and only a limited number of cases describing serrated lesions in both the small and large bowel have been reported. Rubio first described a duodenal sessile serrated lesion associated with familial adenomatous polyposis in 2004 [4]. Subsequent reports have documented additional duodenal serrated lesions, most of which were hyperplastic polyps [5-10].

Notably, Becq et al. reported the first association between SPS and a duodenal serrated lesion demonstrating a shared immunophenotype with colonic lesions, including overexpression of MUC2, MUC5AC, and Annexin A10 at both sites [5]. Despite the importance of these markers, in our case, the diagnosis was based on histomorphological features alone.

In this report, we describe a rare case of SPS with extensive involvement of the gastrointestinal tract, including the duodenum, jejunum, ileum, and colon.

## Case presentation

**Figure 1:**
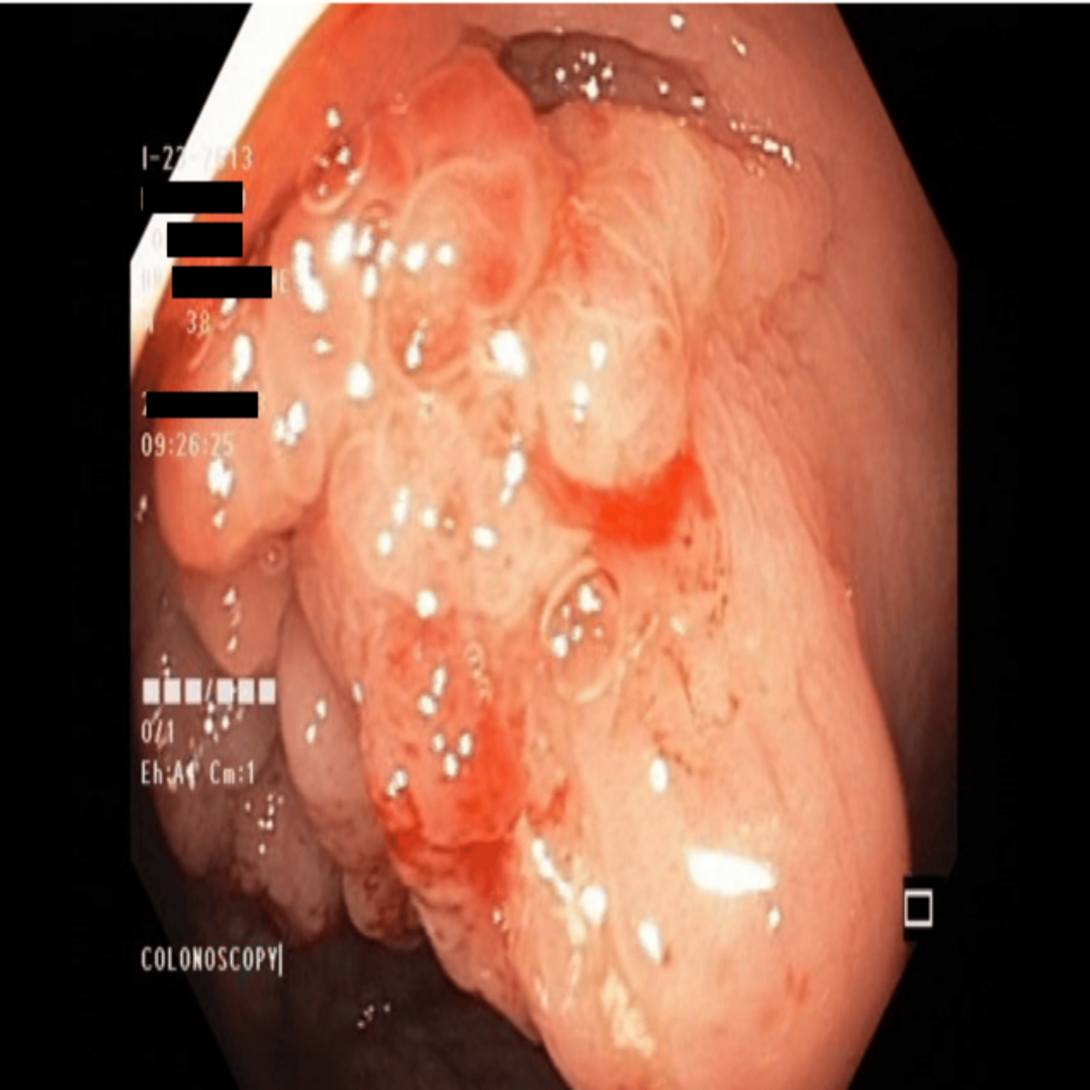
The ileocecal valve showed several polyps, the largest measuring 2 cm.

**Figure 2:**
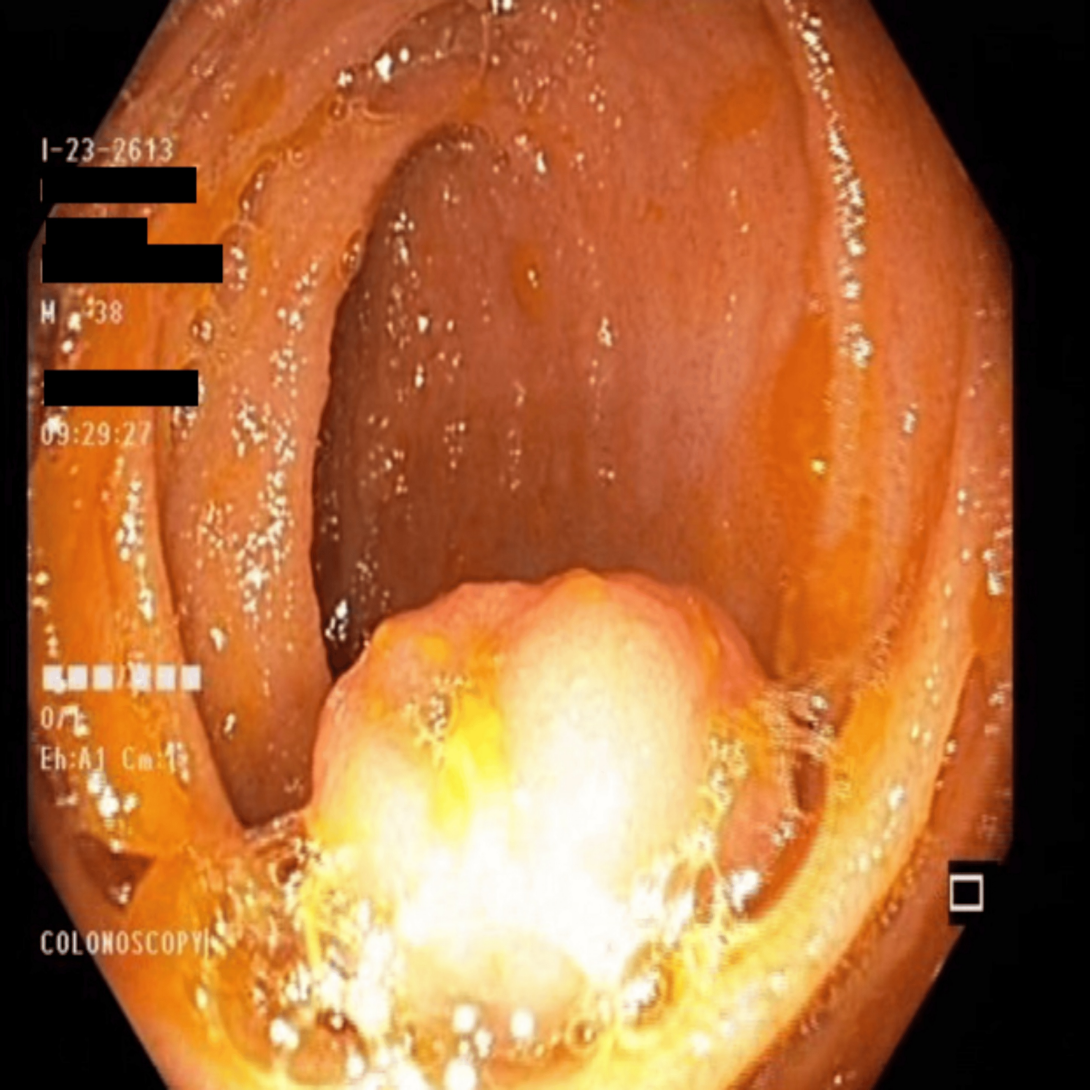
Terminal ileum showing a large polyp.

**Figure 3:**
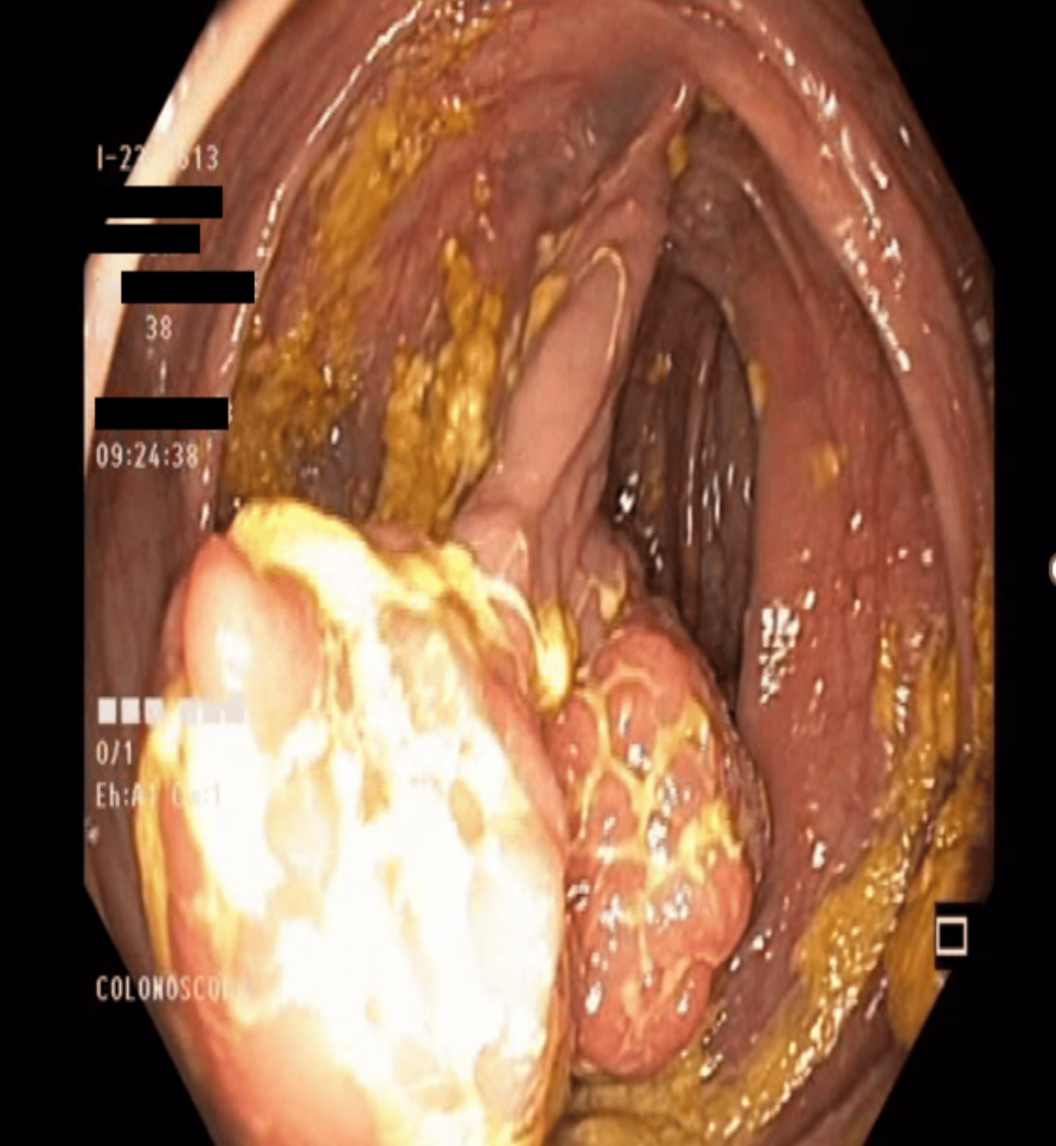
Figure shows erythematous lesions with areas of mucous/exudative covering. The surrounding mucosa appears congested and edematous, with inflammatory changes and adherent debris.

**Figure 4:**
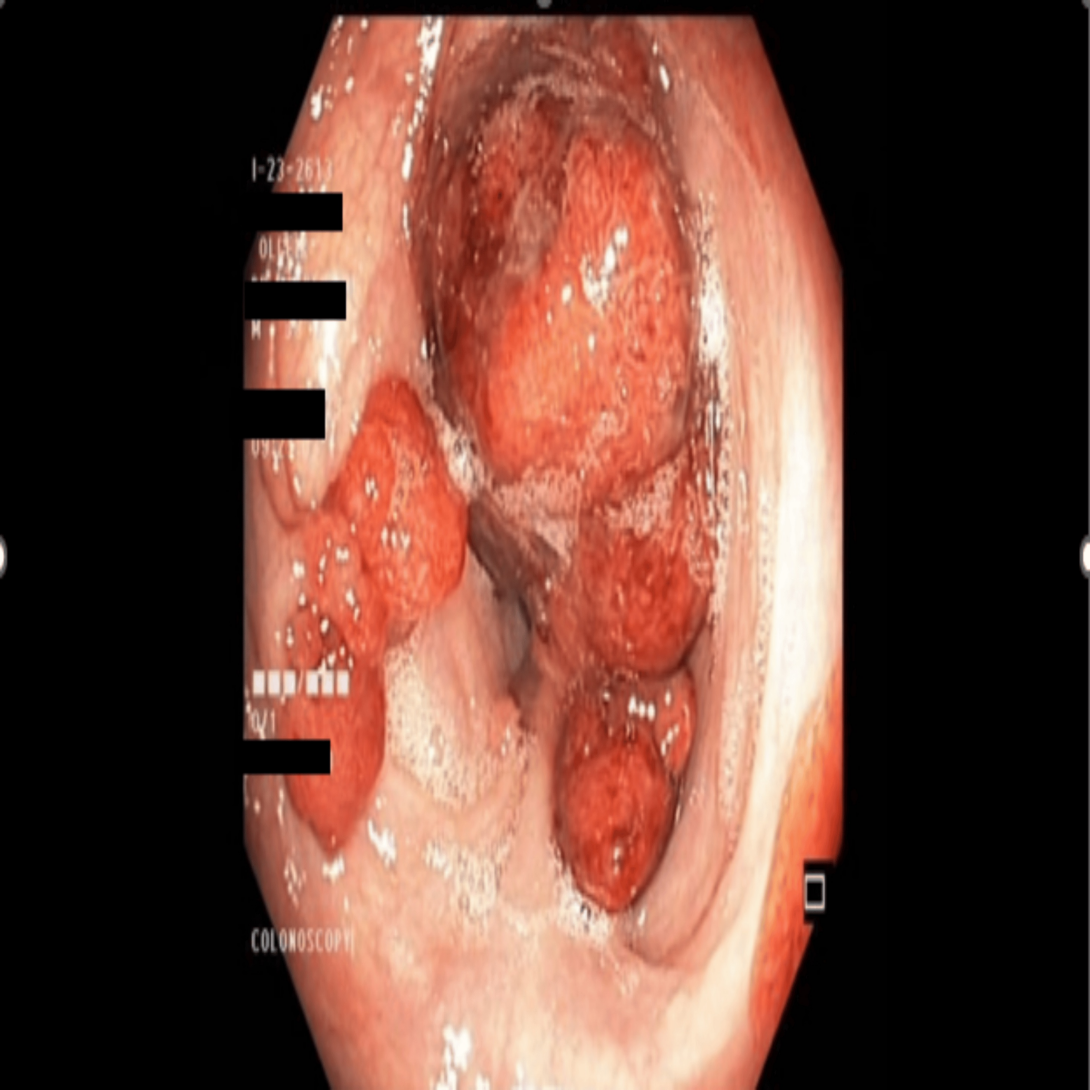
Figure showing several large pedunculated polyps of varying sizes.

**Figure 5:**
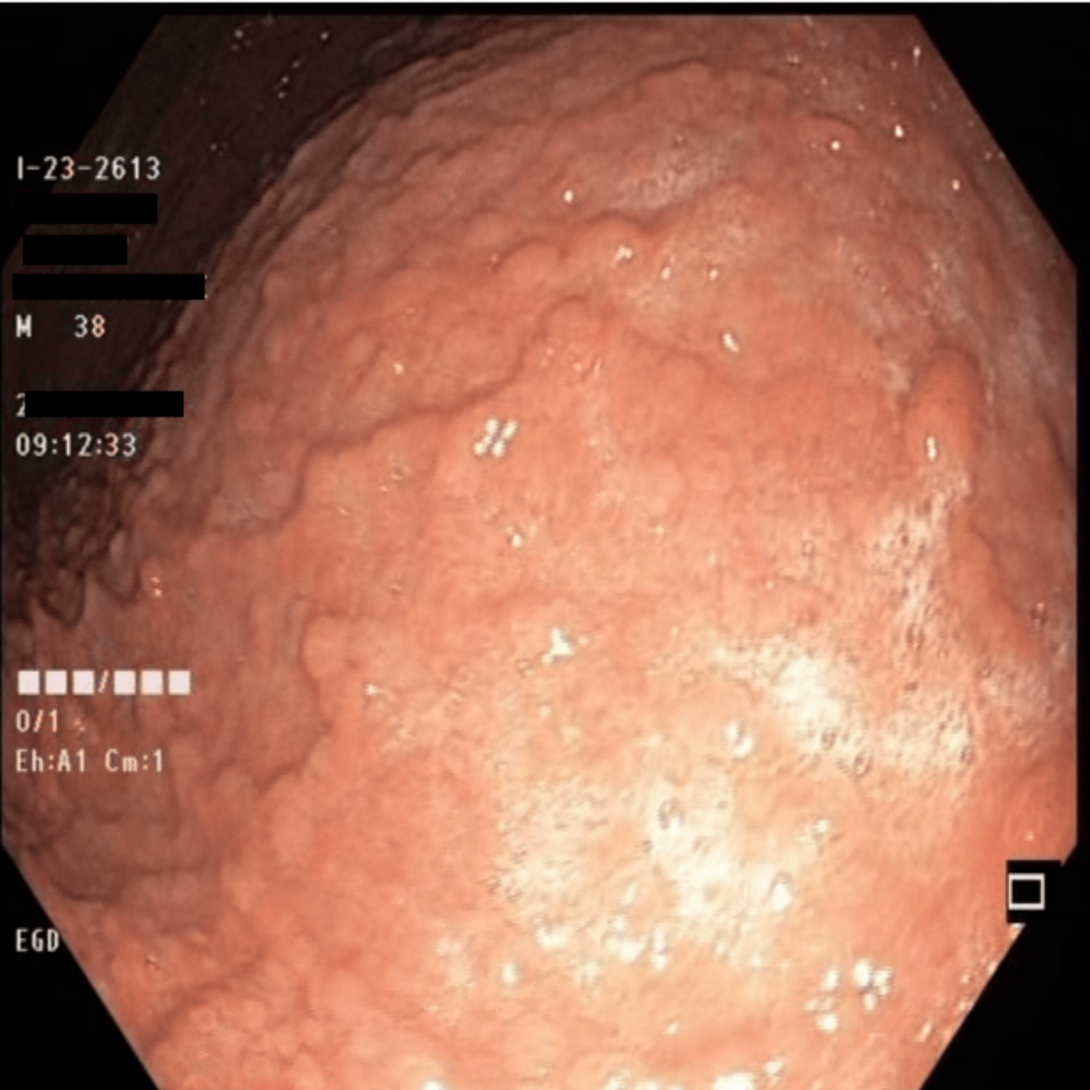
Figure showing gastric mucosa with loss of the normal smooth mucosal pattern, prominent irregular folds, and multiple small elevations.

**Figure 6:**
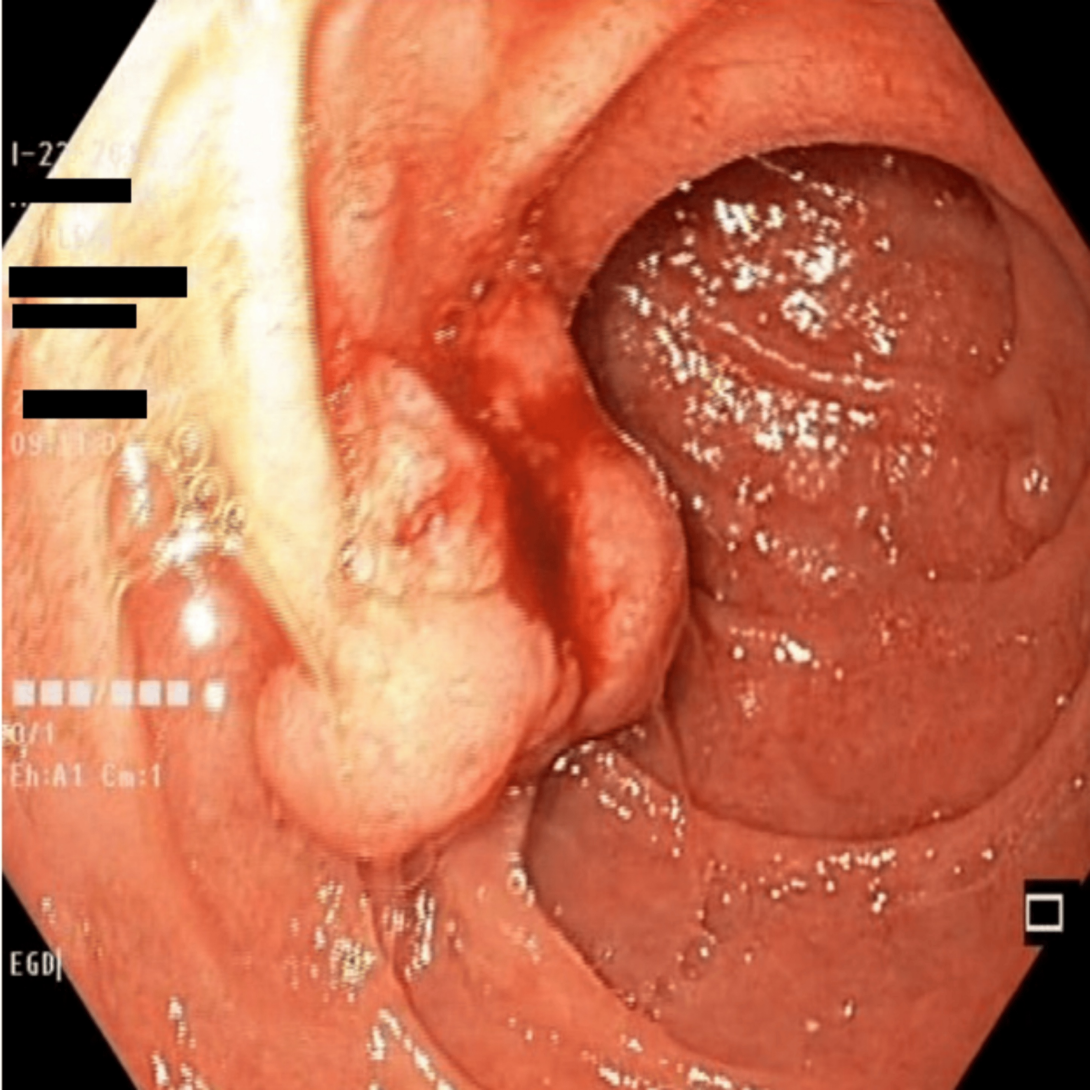
Figure showing two pedunculated duodenal polyps.

**Figure 7:**
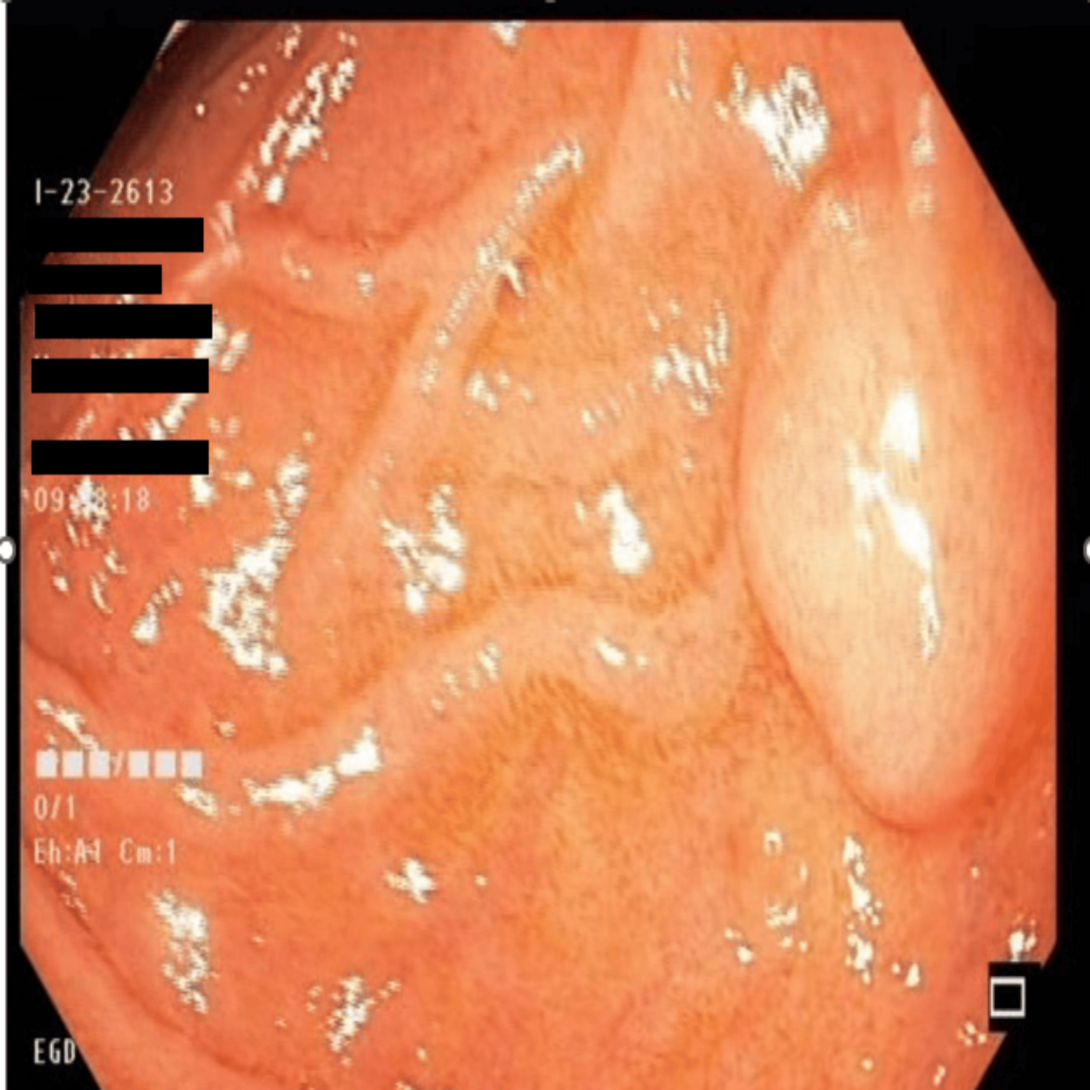
Figure showing an elevated duodenal polyp.

This is a case of a 38-year-old, previously healthy, non-smoker male, who presented in June 2023 for abdominal pain and fever. Investigations, including computed tomography (CT) of the abdomen and pelvis, showed an appendiceal abscess and small bowel intussusception in two areas in the left and middle abdominal quadrants without obstruction, for which percutaneous drainage was done. In August 2023, a CT of the abdomen and pelvis was done as a follow-up after management of an appendix abscess in another radiology center, which showed multiple small and large bowel polyps suggestive of intestinal polyposis syndrome. Colonoscopy and esophagogastroduodenoscopy (EGD) were performed after this significant CT of the abdomen and pelvis to rule out intestinal polyposis syndrome, which showed more than 30 large polyps extending from the ileum to the rectum. EGD also showed two duodenal polyps, the largest measuring 2 cm (Figures 1-7).

**Figure 8:**
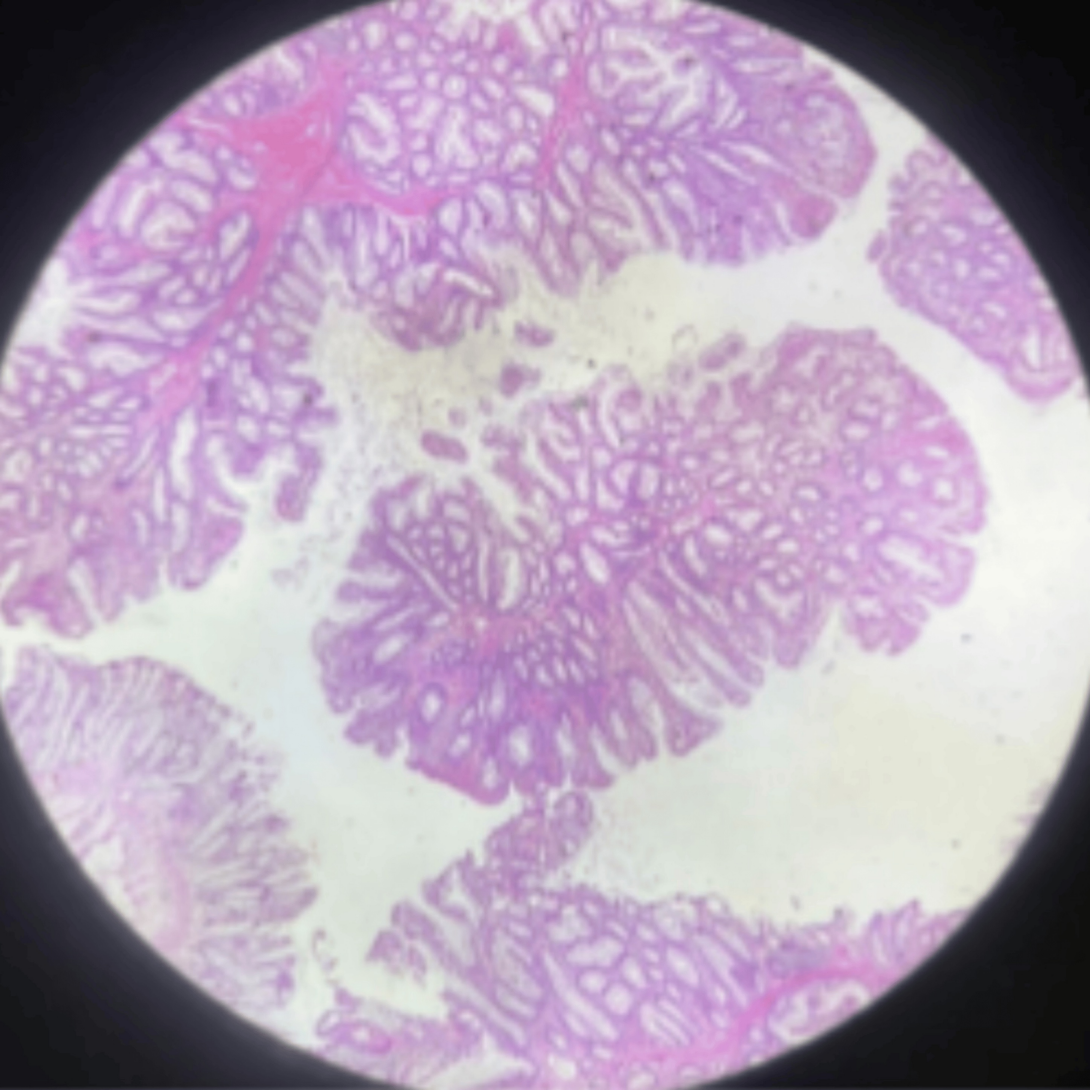
A biopsy specimen of a polyp removed from the colon showing a tubulovillous serrated shape. Hematoxylin and eosin (H&E) stain, low-power magnification (10× eyepiece × 10× objective).

**Figure 9:**
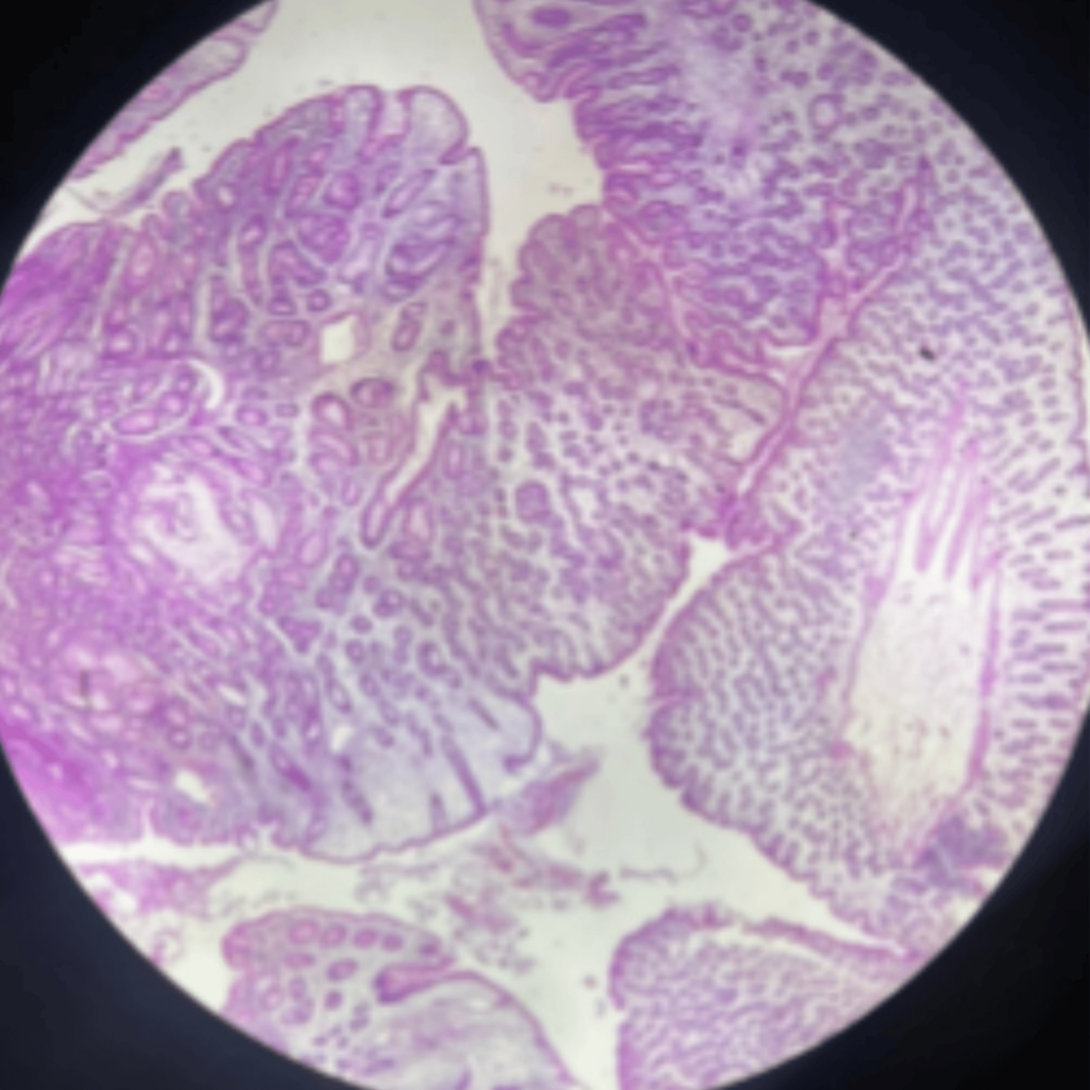
Figure showing a polyp removed from the colon with tubular proliferation. Hematoxylin and eosin (H&E) stain, low-power magnification (10× eyepiece × 10× objective).

**Figure 10:**
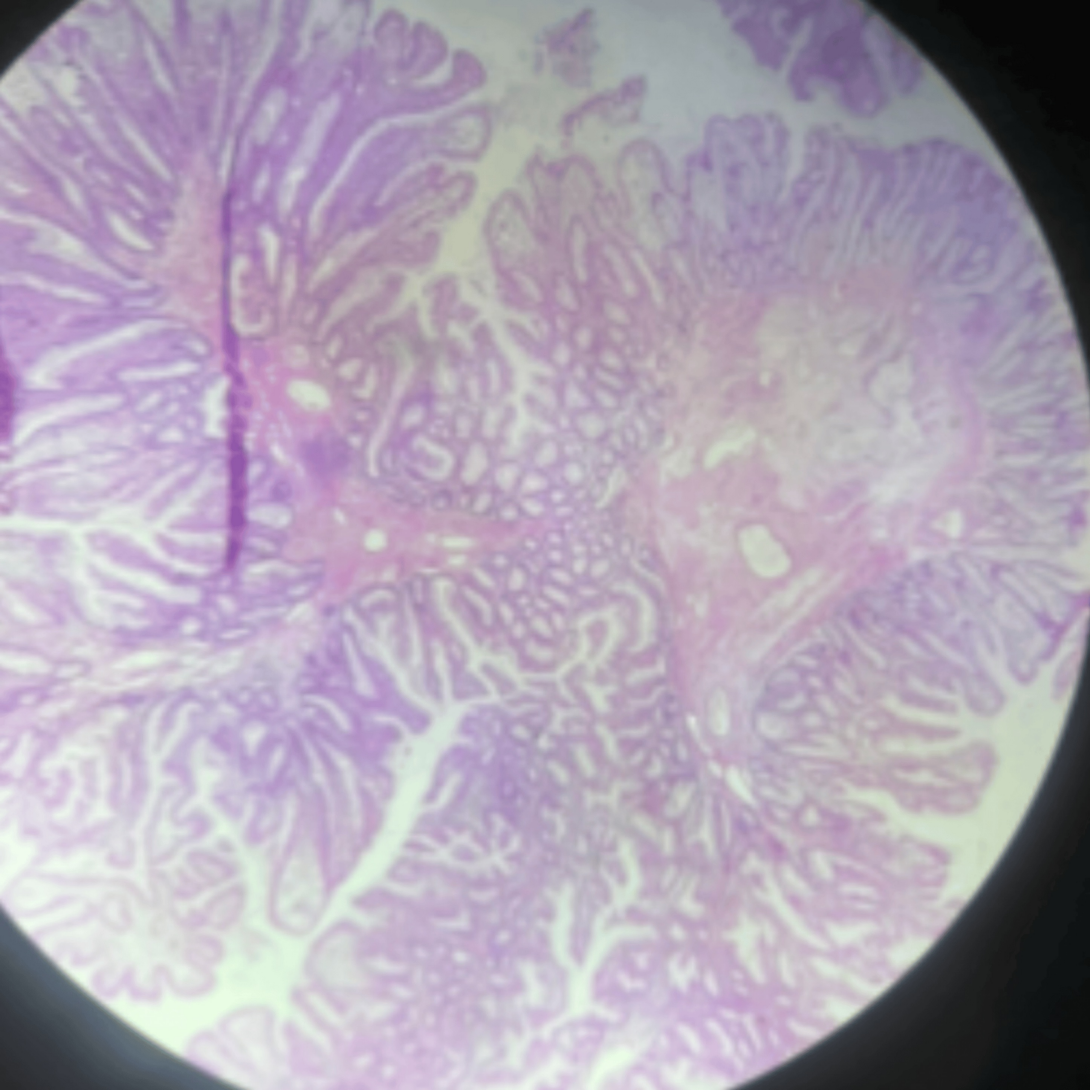
Figure showing a polyp removed from the duodenum with no significant findings. Hematoxylin and eosin (H&E) stain, low-power magnification (10× eyepiece × 10× objective).

Biopsies of the polyps revealed serrated-pattern polyps without dysplasia, except in the duodenum, where no significant findings were detected (Figures 8-10).

**Figure 11:**
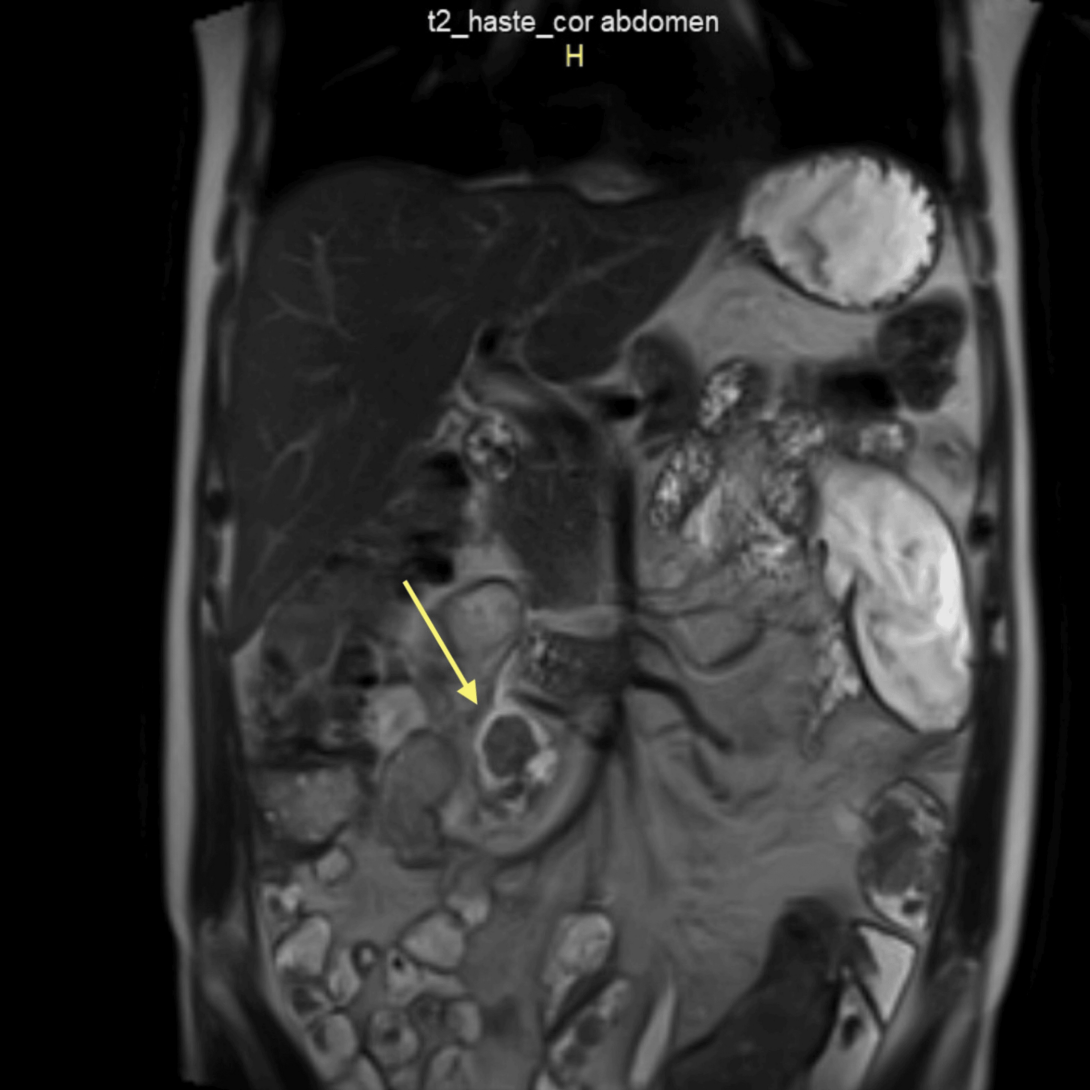
Figure of a magnetic resonance enterography. The yellow arrow marks a large polyp in the small intestine.

**Figure 12:**
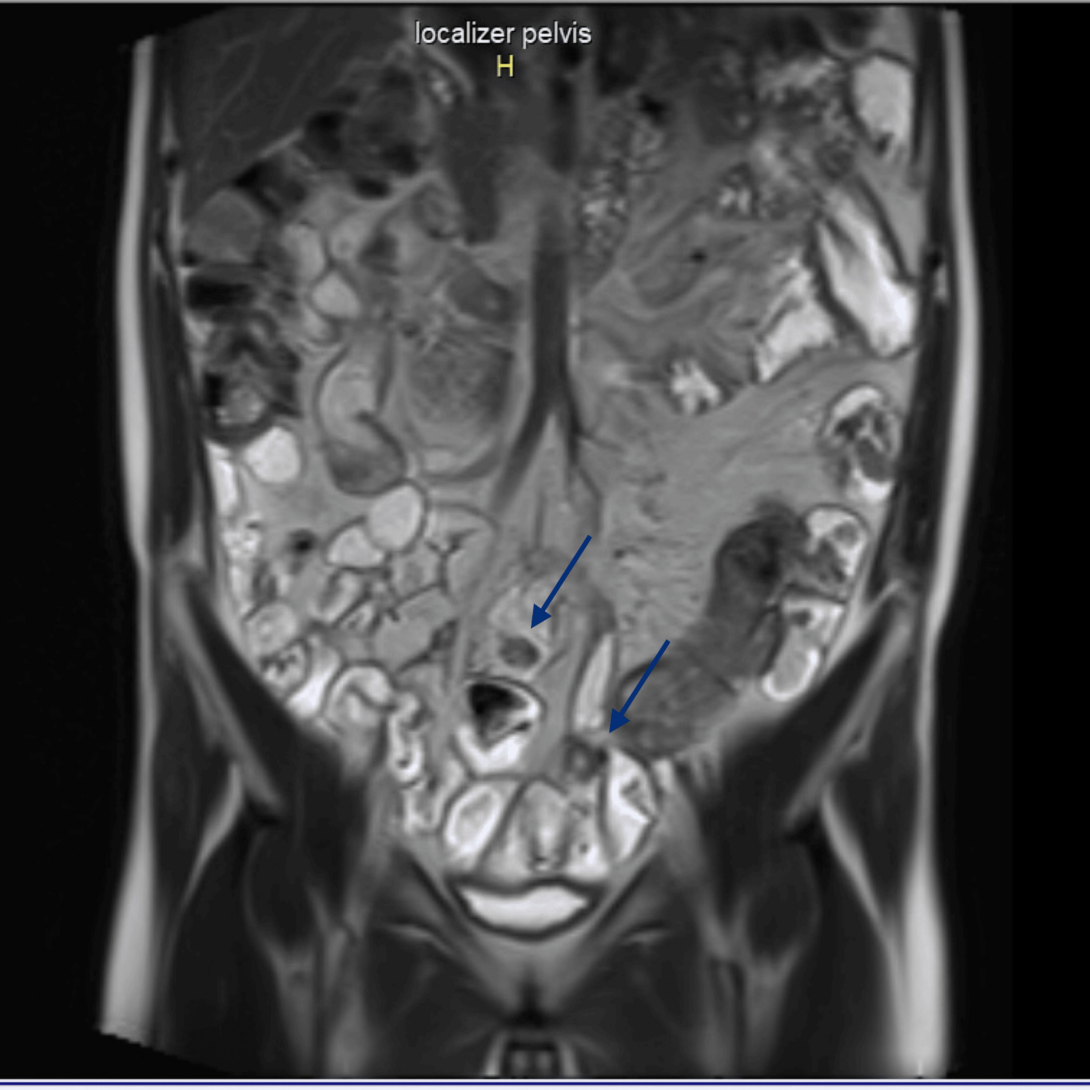
Figure of a magnetic resonance enterography. Blue arrows mark a small intestine polyp.

Magnetic resonance (MR) enterorrhaphy was ordered to further investigate for small bowel polyps and was done in September 2023. It showed an area of intussusception in the jejunum associated with multiple polyps at this level adjacent to each other (Figures 11, 12).

The appendiceal abscesses are potentially associated with the consequences of the polyposis process.

The patient was referred for small bowel resection, especially after developing obstipation in October 2023. Around 1 meter of enterectomy was done, and multiple intestinal polyps were identified during laparotomy, the largest measuring 20 cm from the Treitz ligament, and the pathology results showed serrated polyposis polyps. In February 2024, the patient was scheduled for an elective polypectomy at our institution, during which numerous pedunculated polyps of varying sizes were observed extending from the cecum to 25 cm from the anal verge, many of which were removed using hot and cold snares. In addition, repeat EGD revealed a duodenal polyp, which was also removed. Multiple tubular and tubulovillous adenomas with low-grade dysplasia were identified in the resected colonic polyps. A similar result was obtained from the resected duodenal polyp. After one week, another elective polypectomy session was assigned, aiming to clear the left-sided colon before the elective right colon resection that had been planned after a multidisciplinary decision with the surgery team. Polypectomy was done until the splenic flexure (35 cm from the anal verge), since during the second endoscopic session, removal of left-sided colonic polyps was complicated by persistent active bleeding at approximately 30 cm from the anal verge, which was not controlled despite endoscopic hemostatic measures, including clip application.

**Figure 13:**
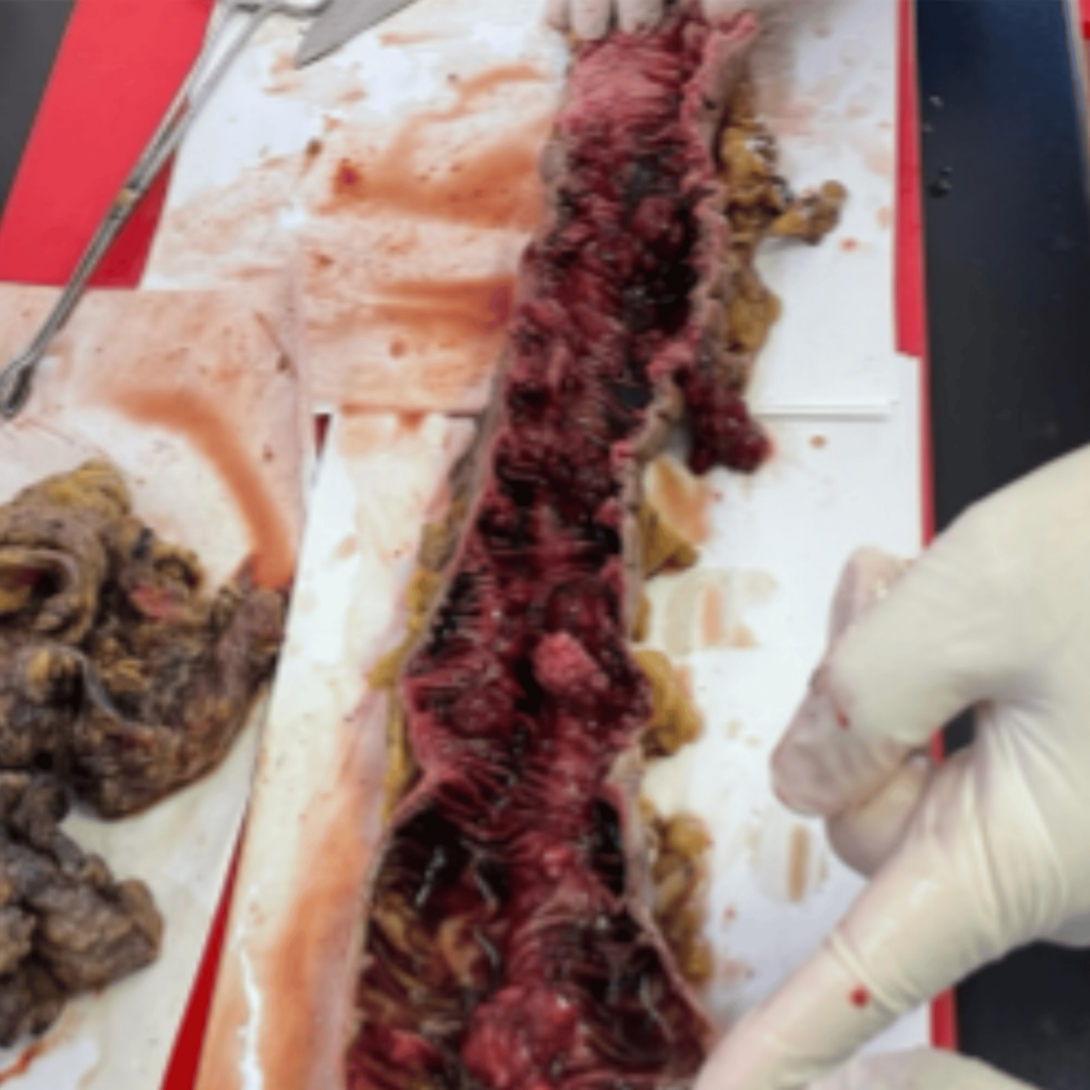
Figure showing gross anatomy revealing two large pedunculated polyps measuring 25 mm and 40 mm in the ascending colon.

**Figure 14:**
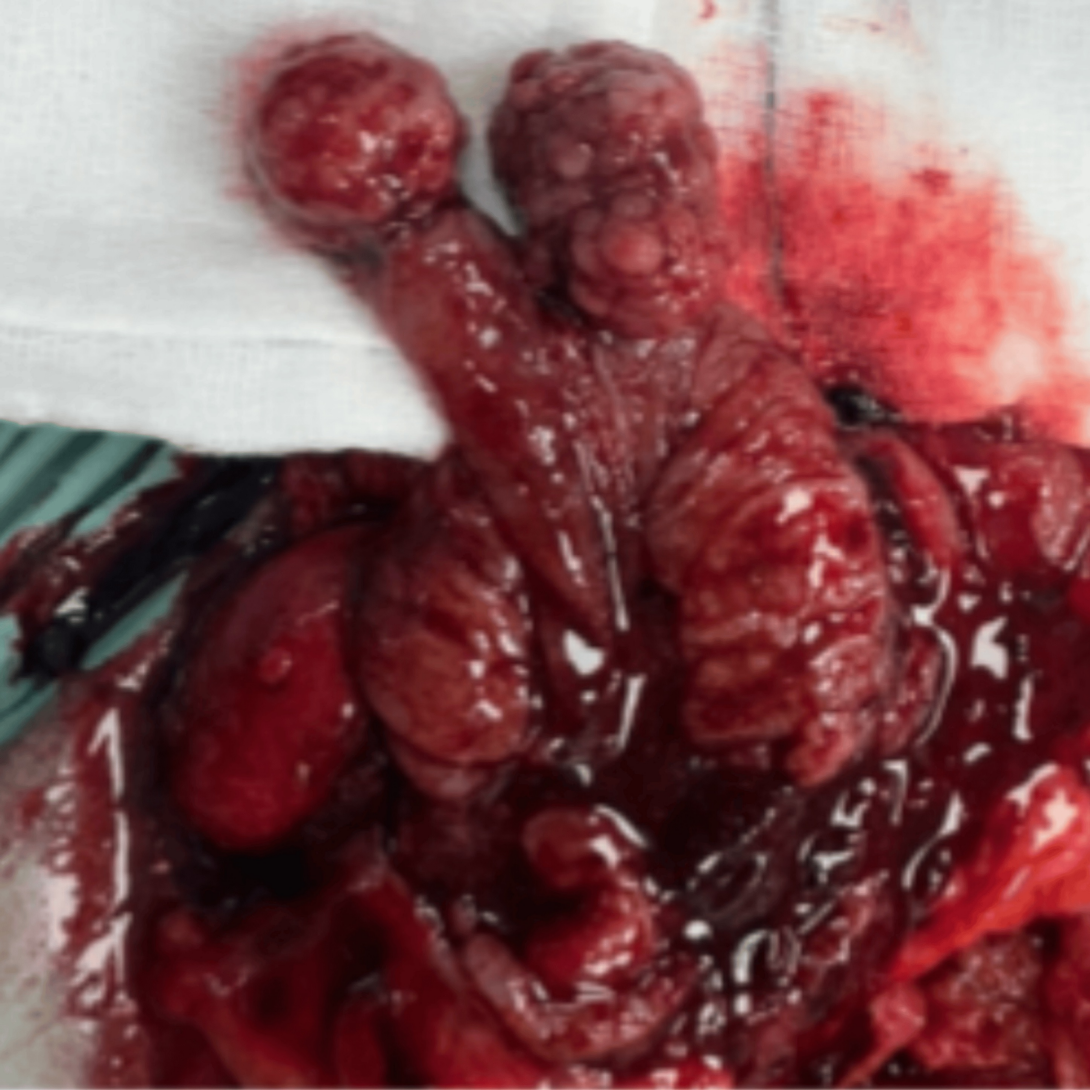
Figure showing gross anatomy of 18 pedunculated polyps ranging in size from 8 mm to 25 mm in the transverse and descending colon.

Given the failure of endoscopic hemostasis, the extensive residual polyp burden, and the risk of recurrent hemorrhage, a multidisciplinary team decision (gastroenterology and colorectal surgery) was made to proceed with urgent subtotal colectomy as a definitive therapeutic and preventive strategy. This approach aimed to control acute bleeding, prevent further life-threatening hemorrhage, and reduce future malignant potential in the context of diffuse polyposis because it has been complicated by an active bleed that has not been controlled with the application of clips (30 cm from the anal verge). Consequently, an urgent subtotal colectomy was done from the distal ileum to the descending colon beyond the site of bleed, and anastomosis was performed between the terminal ileum and the distal descending colon. Resected parts of the colon with the ileocecal valve were sent for pathology. Gross findings revealed two large pedunculated polyps measuring 25 and 40 mm in the ascending colon, and 18 pedunculated polyps ranging from 8 mm to 25 mm in size in the transverse and descending colon (Figures 13, 14).

Microscopically, colonic serrated adenomatous polyposis with cecal diverticular structure was noted, but there was neither wall infiltration nor neoplastic proliferation. In addition, all regional lymph nodes were reactive, and the proximal and distal margins were free. After three months, a colonoscopy was repeated for surveillance of the remaining portion of the colon, and it only showed a few scattered diminutive polyps.

The patient was subsequently scheduled for a surveillance colonoscopy in May 2025, which showed no more polyps or significant findings. Moreover, we advised the patient to encourage his first-degree relatives for screening. We are willing to do genetic testing of the polyps removed from the duodenum, colon, and jejunum to test if the same mutations are present.

Genetic testing is a great addition in this case, but unfortunately, due to the limited resources and financial limitations, it was not performed.

## Discussion

The small intestine accounts for 75% of the total bowel length, yet neoplasms are rare, accounting for about 2% of digestive system cancers [9]. They may arise sporadically or in association with risk factors such as familial adenomatous polyposis (FAP), Crohn’s disease, or celiac sprue [11]. Adenocarcinoma accounts for 30-40% of small intestinal cancers, mainly affecting the duodenum and proximal jejunum [9]. TSAs may behave aggressively, with reported progression to invasive carcinoma in up to 53.4% of cases in a series of 73 lesions [12]. This suggests a malignant potential, possibly higher than that of conventional adenomas, which less frequently show high-grade dysplasia [12]. TSA-associated duodenal adenocarcinoma has been reported and treated endoscopically using EGD [13].

Due to malignant potential, the WHO recommends removal of polyps ≥3 mm at three- to six-month intervals until complete clearance [14]. For surveillance, colonoscopy is recommended every one to two years, or annually in high-risk cases such as SPs ≥10 mm, dysplastic SPs, history of CRC, advanced adenomas, TSAs, or multiple (≥10) serrated lesions/adenomas [11].

Patients with SPS require screening of first-degree relatives. If no colorectal cancer is present, screening begins at the same age as diagnosis; if cancer is present, screening starts 10 years earlier [15]. Surveillance is then repeated every five years if negative, or every one to three years if polyps are detected [15]. Treatment includes endoscopic or surgical approaches. Small sessile serrated lesions (SSLs) are usually removed by cold snare polypectomy, while larger lesions may require endoscopic mucosal resection. In a study of 251 large SSLs (≥10 mm), this method showed low recurrence (<4%) and no major complications [16].

SPS is rare and often difficult to diagnose due to flat lesions and initial misclassification, requiring repeated endoscopic and histological evaluation. Patients with SSLs have an increased risk of colorectal neoplasia, so regular surveillance and careful pathological review are essential.

## Conclusions

SPS is an underrecognized condition, yet it is a clinically significant condition associated with an increased risk of CRC. This case highlights the rare involvement of both the small and large intestines, emphasizing the importance of evaluation in this condition beyond the colon. Early detection, regular surveillance, and timely intervention, whether endoscopic or surgical, are essential to prevent malignant transformation and improve patient outcomes.
